# How to make the most of NE dictionaries in statistical NER

**DOI:** 10.1186/1471-2105-9-S11-S5

**Published:** 2008-11-19

**Authors:** Yutaka Sasaki, Yoshimasa Tsuruoka, John McNaught, Sophia Ananiadou

**Affiliations:** 1 School of Computer Science, University of Manchester, 131 Princess Street, Manchester, M1 7DN, UK; 2 National Centre for Text Mining, Manchester Interdisciplinary Biocentre, 131 Princess Street, Manchester, M1 7DN, UK

## Abstract

**Background:**

When term ambiguity and variability are very high, dictionary-based *Named Entity Recognition *(*NER*) is not an ideal solution even though large-scale terminological resources are available. Many researches on statistical NER have tried to cope with these problems. However, it is not straightforward how to exploit existing and additional *Named Entity *(*NE*) dictionaries in statistical NER. Presumably, addition of NEs to an NE dictionary leads to better performance. However, in reality, the retraining of NER models is required to achieve this. We chose protein name recognition as a case study because it most suffers the problems related to heavy term variation and ambiguity.

**Methods:**

We have established a novel way to improve the NER performance by adding NEs to an NE dictionary without retraining. In our approach, first, known NEs are identified in parallel with *Part-of-Speech *(*POS*) tagging based on a general word dictionary and an NE dictionary. Then, statistical NER is trained on the POS/PROTEIN *tagger outputs *with correct NE labels attached.

**Results:**

We evaluated performance of our NER on the standard JNLPBA-2004 data set. The F-score on the test set has been improved from 73.14 to 73.78 after adding protein names appearing in the training data to the POS tagger dictionary without any model retraining. The performance further increased to 78.72 after enriching the tagging dictionary with test set protein names.

**Conclusion:**

Our approach has demonstrated high performance in protein name recognition, which indicates how to make the most of known NEs in statistical NER.

## Background

The accumulation of online biomedical information has been growing at a rapid pace, mainly attributed to a rapid growth of a wide range of repositories of biomedical data and literature. The automatic construction and update of scientific *knowledge bases *is a major research topic in Bioinformatics. One way of populating these knowledge bases is through *named entity recognition *(*NER*). Unfortunately, biomedical NER faces many problems, e.g., protein names are extremely difficult to recognize due to high ambiguity and variability. A further problem in protein name recognition arises at the tokenization stage. Some protein names include punctuation or special symbols, which may cause tokenization to lose some word concatenation information in the original sentence. For example, protein IL-2 and mathematical expression IL – 2 fall into the same token sequence IL – 2 as usually dash (or hyphen) is designated as a token delimiter. In this sense, protein name recognition from tokenized sequence is more challenging than that from text.

Research into NER is centered around three approaches: dictionary-based, rule-based and machine learning-based approaches [1]. To overcome the usual NER pitfalls, we have opted for a hybrid approach combining dictionary-based and machine learning approaches, which we call *dictionary-based statistical NER approach*. After identifying protein names in text, we link these to semantic identifiers, such as UniProt accession numbers. In this paper, we focus on the evaluation of our dictionary-based statistical NER.

## Methods

Our dictionary-based statistical approach consists of two components: dictionary-based POS/PROTEIN tagging and statistical sequential labelling. First, dictionary-based POS/PROTEIN tagging finds candidates for protein names using a dictionary. The dictionary maps strings to parts of speech (POS), where the POS tag-set is augmented with a tag NN-PROTEIN. Then, sequential labelling applies to reduce false positives and false negatives in the POS/PROTEIN tagging results. Expandability is supported through allowing a user of the NER tool to improve NER coverage by adding NE entries to the dictionary. In our approach, retraining of models is not required after dictionary enrichment.

Recently, *Conditional Random Fields *(*CRFs*) have been successfully applied to sequence labelling problems, such as POS tagging and NER. [2] The main idea of CRFs is to estimate a conditional probability distribution over label sequences, rather than over local directed label sequences as with Hidden Markov Models [3] and Maximum Entropy Markov Models [4]. Parameters of CRFs can be efficiently estimated through the log-likelihood parameter estimation using the forward-backward algorithm, a dynamic programming method.

### Training and test data

Experiments were conducted using the training and test sets of the JNLPBA-2004 data set [5].

#### Training data

The training data set used in JNLPBA-2004 is a set of tokenized sentences with manually annotated term class labels. The sentences are taken from the Genia corpus (version 3.02) [6], in which 2,000 abstracts were manually annotated by a biologist, drawing on a set of POS tags and 36 biomedical term classes. In the JNLPBA-2004 shared task, performance in extracting five term classes, i.e., protein, DNA, RNA, cell line, and cell type classes, were evaluated.

#### Test data

The test data set used in JNLPBA-2004 is a set of tokenized sentences extracted from 404 separately collected MEDLINE abstracts, where the term class labels were manually assigned, following the annotation specification of the Genia corpus.

### Overview of dictionary-based statistical NER

**Figure 1:**
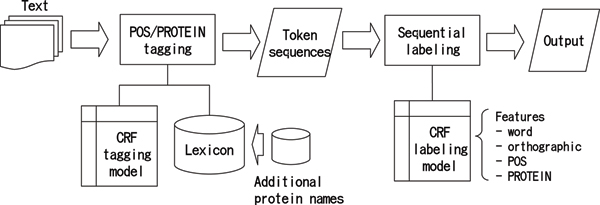
Block diagram of lexicon-based statistical NER.

Figure 1 shows the block diagram of dictionary-based statistical NER. Raw text is analyzed by a POS/PROTEIN tagger based on a CRF tagging model and dictionary, and then converted into token sequences. Strings in the text that match with protein names in the dictionary will be tagged as NN-PROTEIN depending on the context around the protein names. Since it is not realistic to enumerate all protein names in the dictionary, due to their high variability of form, instead previously unseen forms are predicted to be protein names by statistical sequential labelling. Finally, protein names are identified from the POS/PROTEIN tagged token sequences via a CRF labelling model.

**Figure 2:**
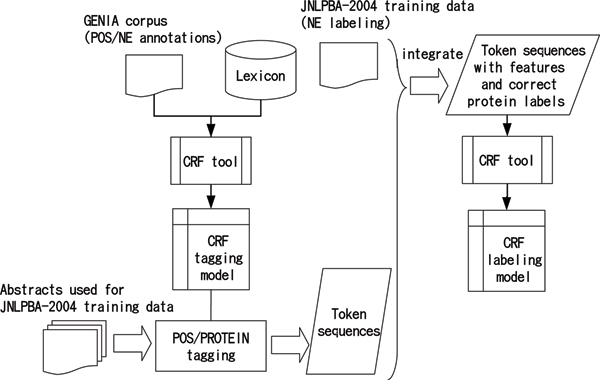
Block diagram of tagging and labelling model generation.

Figure 2 shows the block diagram of the training procedure for both POS/PROTEIN tagging and sequential labelling. The tagging model is created using the Genia corpus (version 3.02) and a dictionary. Using the tagging model, MEDLINE abstracts used for the JNLPBA-2004 training data set are then POS/PROTEIN-tagged. The output token sequences over these abstracts are then integrated with the correct protein labels of the JNLPBA-2004 training data. This process results in the preparation of token sequences with features and correct protein labels. A CRF labelling model is finally generated by applying a CRF tool to these decorated token sequences.

#### Dictionary-based POS/PROTEIN tagging

The dictionary-based approach is beneficial when a sentence contains some protein names that conflict with general English words. Otherwise, if the POS tags of sentences are decided without considering possible occurrences of protein names, POS sequences could be disrupted. For example, in "met proto-oncogene precursor", *met *might be falsely recognized as a verb by a non dictionary-based tagger.

Given a sentence, the dictionary-based approach extracts protein names as follows. Find all word sequences that match the lexical entries, and create a token graph (i.e., *trellis*) according to the word order. Estimate the score of every path using the weights of node and edges estimated by training using Conditional Random Fields. Select the best path.

**Figure 3:**
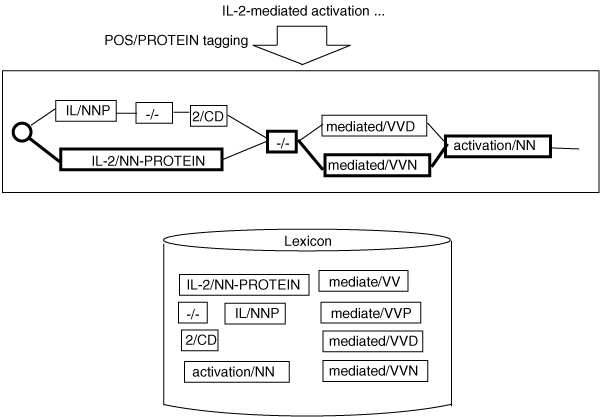
Example of lexicon-based POS/Protein tagging.

Figure 3 shows an example of our dictionary-based approach. Suppose that the input is "IL-2-mediated activation". A trellis is created based on the lexical entries in a dictionary. The selection criteria for the best path are determined by the CRF tagging model trained on the Genia corpus. In this example, IL-2/NN-PROTEIN -/- mediated/VVN activation/NN is selected as the best path. Following Kudo et al. [7], we adapted the core engine of the CRF-based morphological analyzer, MeCab [8], to our POS/PROTEIN tagging task. MeCab's dictionary databases employ double arrays [9] which enable efficient lexical look-ups.

The features used were:

• POS

• PROTEIN

• POS-PROTEIN

• bigram of adjacent POS

• bigram of adjacent PROTEIN

• bigram of adjacent POS-PROTEIN

During the construction of the trellis, white space is considered as the delimiter unless otherwise stated within dictionary entries. This means that unknown tokens are character sequences without spaces.

#### Dictionary construction

A dictionary-based approach requires the dictionary to cover not only a wide variety of biomedical terms but also entries with:

• all possible capitalization

• all possible linguistic inflections

We constructed a freely available, wide-coverage English word dictionary that satisfies these conditions. We did consider the MedPost pos-tagger package [10] which contains a free dictionary that has downcased English words; however, this dictionary is not well curated as a dictionary and the number of entries is limited to only 100,000, including inflections.

Therefore, we started by constructing an English word dictionary. Eventually, we created a dictionary with about 266,000 entries for English words (systematically covering inflections) and about 1.3 million entries for protein names.

We created the general English part of the dictionary from WordNet by semi-automatically adding POS tags. The POS tag set is a minor modification of the Penn Treebank POS tag set [11], in that protein names are given a new POS tag, NN-PROTEIN. Further details on construction of the dictionary now follow.

**Protein names **were extracted from the BioThesaurus [12]. After selecting only those terms clearly stated as protein names, pair-wise distinct 1,341,992 protein names in total were added to the dictionary.

**Nouns **were extracted from WordNet's noun list. Words starting with lower case and upper case letters were determined as NN and NNP, respectively. Nouns in NNS and NNPS categories were collected from the results of POS tagging articles from Plos Biology Journal [13] with TreeTagger [14].

**Verbs **were extracted from WordNet's verb list. We manually curated VBD, VBN, VBG and VBZ verbs with irregular inflections based on WordNet. Next, VBN, VBD, VBG and VBZ forms of regular verbs were automatically generated from the WordNet verb list.

**Adjectives **were extracted from WordNet's adjective list. We manually curated JJ, JJR and JJS of irregular inflections of adjectives based on the WordNet irregular adjective list. Base form (JJ) and regular inflections (JJR, JJS) of adjectives were also created based on the list of adjectives.

**Adverbs **were extracted from WordNet's adverb list. Both the original and capitalized forms were added as RB.

**Pronouns **were manually curated. PRP and PRP$ words were added to the dictionary.

**Wh-words **were manually curated. As a result, WDT, WP, WP$ and WRB words were added to the dictionary.

**Words for other parts of speech **were manually curated.

#### Statistical prediction of protein names

Statistical sequential labelling was employed to improve the coverage of protein name recognition and to remove false positives resulting from the previous stage (dictionary-based tagging).

We used the JNLPBA-2004 training data, which is a set of tokenized word sequences with IOB2 [15] protein labels. As shown in Figure 2, POSs of tokens resulting from tagging and tokens of the JNLPBA-2004 data set are integrated to yield training data for sequential labelling. During integration, when the single token of a protein name found after tagging corresponds to a sequence of tokens from JNLPBA-2004, its POS is given as NN-PROTEIN1, NN-PROTEIN2,..., according to the corresponding token order in the JNLPBA-2004 sequence.

Following the data format of the JNLPBA-2004 training set, our training and test data use the IOB2 labels, which are "B-protein" for the first token of the target sequence, "I-protein" for each remaining token in the target sequence, and "O" for other tokens. For example, "Activation of the IL 2 precursor provides" is analyzed by the POS/PROTEIN tagger as follows.

Activation     NN

of             IN

the            DT

IL 2 precursor NN-PROTEIN

provides       VVZ

The tagger output is given IOB2 labels as follows:

Activation NN 0

of         IN 0

the        DT 0

IL         NN-PROTEIN1 B-PROTEIN

2          NN-PROTEIN2 I-PROTEIN

precursor  NN-PROTEIN3 I-PROTEIN

provides   VVZ 0

We used CRF models to predict the IOB2 labels. The following features were used in our experiments.

• word feature

• orthographic features

- the first letter and the last four letters of the word form, in which capital letters in a word are normalized to "A", lower case letters are normalized to "a", and digits are replaced by "0", *e.g*., the word form of IL-2 is AA-0.

- postfixes, the last two and four letters

• POS feature

• PROTEIN feature

The window size was set to ± 2 of the current token.

## Results and discussion

### Protein name recognition performance

Table 1 shows our protein name recognition results, showing the differential effect of various combinations of strategies. Results are expressed according to recall (R), precision (P), and F-measure (F), which here measure how accurately our various experiments determined the left boundary (Left), the right boundary (Right), and both boundaries (Full) of protein names. The baseline for tagging (row (a)) shows the protein name detection performance of our dictionary-based tagging using our large protein name dictionary, where no training for protein name prediction was involved. The F-score of this baseline tagging method was 47.96.

**Table 1:** Protein name recognition performance

Tagging		R	P	F
(a) POS/PROTEIN tagging	Full	52.91	43.85	47.96
	Left	61.48	50.95	55.72
	Right	61.38	50.87	55.63

Sequential Labelling		R	P	F

(b) Word feature	Full	63.23	70.39	66.62
	Left	68.15	75.86	71.80
	Right	69.88	77.79	73.63

(c) (b) + orthographic feature	Full	77.17	67.52	72.02
	Left	82.51	72.20	77.01
	Right	84.29	73.75	78.67

(d) (c) + POS feature	Full	76.46	68.41	72.21
	Left	81.94	73.32	77.39
	Right	83.54	74.75	78.90

(e) (d) + PROTEIN feature	Full	77.58	69.18	73.14
	Left	82.69	73.74	77.96
	Right	84.37	75.24	79.54

(f) (e) after adding protein names in the training set to the lexicon	Full	**79.85**	**68.58**	**73.78**
	Left	84.82	72.85	78.38
	Right	86.60	74.37	80.02

Protein name recognition performance of the proposed method, evaluated by recall (R), precision (P), and F-measure (F). The left boundary (Left), the right boundary (Right), and both boundary (Full) recognition performance were measured. (a) the performance of POS/PROTEIN tagging. (b) the performance of sequential labelling when using the word feature only. (c) the performance of sequential labelling when using the word and orthographic features. (d) the performance of sequential labelling when using the word, orthographic, and POS features. (e) the performance of sequential labelling when using the word, orthographic, POS, and PROTEIN name features. (f) the performance of sequential labelling with the features used in (e) after adding protein names appearing in the *training set *to the lexicon. NB: no retraining was conducted.

The baseline for sequential labelling (row (b)) shows the prediction performance when using only word features where no orthographic and POS features were used. The F-score of the baseline labelling method was 66.62. When orthographic feature was added (row (c)), the F-score increased by 5.40 to 72.02. When the POS feature was added (row (d)), the F-score increased by 0.19 to 72.21. Using all features (row (e)), the F-score reached 73.14. Surprisingly, adding protein names appearing in the *training data *to the dictionary further improved the F-score by 0.64 to 73.78, which is a state-of-the-art performance in protein name recognition using the JNLPBA-2004 data set.

Tagging and labelling speeds were measured using an unloaded Linux server with quad 1.8 GHz Opteron cores and 16 GB memory. The dictionary-based POS/PROTEIN tagger is very fast even though the total size of the dictionary is more than one million. The processing speed for tagging and sequential labelling of the 4,259 sentences of the test set data took 0.3 sec and 7.3 sec, respectively, which means that in total it took 7.6 sec. for recognizing protein names in the plain text of 4,259 sentences.

### Dictionary enrichment

The advantage of the dictionary-based statistical approach is that it is versatile, as the user can easily improve its performance with no retraining. We assume the following situation as the ideal case: suppose that a user needs to analyze a large amount of text with protein names. The user wants to know the maximum performance achievable for identifying protein names with our dictionary-based statistical recognizer which can be achieved by adding more protein names to the current dictionary. Note that protein names should be identified in context. That is, recall of the NER results with the ideal dictionary is not 100%. Some protein names in the ideal dictionary are dropped during statistical tagging or labelling.

Table 2 shows the scores after each step of dictionary enrichment. The first block (Tagging) shows the tagging performance after adding protein names appearing in the *test set *to the dictionary. The second block (Labelling) shows the performance of the sequence labelling of the output of the first step. Note that tagging and the sequence labelling models are not retrained using the test set.

**Table 2:** Upper bound protein name recognition performance after ideal lexicon enrichment

Method		R	P	F
Tagging (+test set protein names)	Full	79.02	61.87	69.40
	Left	82.28	64.42	72.26
	Right	80.96	63.38	71.10

Labelling (+test set protein names)	full	**86.13**	**72.49**	**78.72**
	Left	89.58	75.40	81.88
	Right	90.23	75.95	82.47

The upper bound performance on the JNLPBA-2004 test set by enriching the lexicon with protein names appearing in the *test set*. NB: It was the only the lexicon that was modified. The tagging and sequential labelling models were not retrained using the test set. The first block shows the performance of POS/PROTEIN tagging after adding protein names appearing in the test set to the dictionary. Since many protein names overlap with general English words, sometimes protein names in sentences are not recognized as protein names. The second block shows the performance of the sequence labelling based on the tagging output. Note that the tagging and sequential labelling models were *not *retrained using the test set.

## Discussion

It is not possible in reality to train the recognizer on target data, *i.e.*, the test set, but it would be possible for users to add discovered protein names to the dictionary so that they could improve the overall performance of the recognizer without retraining.

Rule-based and procedural approaches are taken in [16,17]. Machine learning-based approaches are taken in [18-24]. Machine learning algorithms used in these studies are Naive Bayes, C4.5, Maximum Entropy Models, Support Vector Machines, and Conditional Random Fields. Most of these studies applied machine learning techniques to *tokenized *sentences.

Table 3 shows the scores reported by other systems. While the difference in the performance between our system and other top 5 systems is not statistically significant, it is important that our approach achieved state-of-the-art performance, which justify our approach, and that the performance increased by adding NE entries to the dictionary.

**Table 3:** Conventional results for protein name recognition

Authors	R	P	F
Tsai et al. [25]	71.31	79.36	75.12
**Our system**	**79.85**	**68.58**	**73.78**
Zhou and Su [26]	69.01	79.24	73.77
Kim and Yoon [27]	75.82	71.02	73.34
Okanohara et al. [21]	77.74	68.92	73.07
Tsuruoka [23]	81.41	65.82	72.79
Finkel et al. [28]	77.40	68.48	72.67
Settles [29]	76.1	68.2	72.0
Song et al. [32]	65.50	73.04	69.07
Rössler [30]	72.9	62.0	67.0
Park et al. [31]	69.71	59.37	64.12

Conventional scores on the test set of JNLPBA-2004 shared task.

Tsai et al. [25] and Zhou and Su [26] combined machine learning techniques and manual heuristics tailored for the data set. Tsai et al. [25] applied CRFs to the JNLPBA-2004 data. After applying pattern-based post-processing, they achieved the best F-score (75.12) among those reported so far. Kim and Yoon [27] also applied heuristic post-processing. Because of the domain dependence of their NER methods, when porting a NER system to a new domain (e.g., metabolite names), the developer of the NER system, not a user, currently needs to devise new post-processing heuristics for the new domain to outperform purely principled methods.

The GENIA Tagger [23] is trained on the JNLPBA-2004 Corpus. Okanohara et al. [21] employed semi-Markov CRFs whose performance was evaluated against the JNLPBA-2004 data set. Yamamoto et al. [24] used SVMs for character-based protein name recognition and sequential labelling. Their protein name extraction performance was 69%. Finkel et al. [28] employed MEMM. CRFs are applied in Settles [29]. Rössler [30] and Park et al. [31] applied SVMs while Song et al. [32] applied both SVMs and CRFs.

This paper extends the machine learning approach with a curated dictionary and CRFs and achieved high F-score 73.78. Table 4 shows typical recognition errors found in the recognition results that achieved F-score 73.78. It is one of the reasons for the recognition errors that the data set contains general protein names, such as domain, family, and binding site names as well as anaphoric expressions, which are usually not covered by protein name repositories. In some cases, protein name annotations of the Genis Corpus, hence JNLPBA-2004 data set, are not consistent. Therefore, our impression on the performance, i.e., an F-score of 73.78, is that the recognition quality is sufficiently high.

**Table 4:** Error analysis

	False positives		
	Cause	Correct extraction	Identified term

1	lexicon	-	protein, binding sites
2	prefix word	trans-acting factor	common trans-acting factor
3	unknown word	-	ATTTGCAT
4	sequential labelling error	-	additional proteins
5	test set error	-	Estradiol receptors

	False negatives		

	Cause	Correct extraction	Identified term

1	anaphoric	(*the*) receptor, (*the*) binding sites	-
2	coordination (and, or)	transcription factors NF-kappa B and AP-1	transcription factors NF-kappa B
3	prefix word	activation protein-1	protein-1
		catfish STAT	STAT
4	postfix word	nuclear factor kappa B complex	nuclear factor kappa B
5	plural	protein tyrosine kinase(s)	protein tyrosine kinase
6	family name, biding site, and domain	T3 binding sites	-
		residues 639–656	-
7	sequential labelling error	PCNA	-
		Chloramphenicol acetyltransferase	-
8	test set error	superfamily member	-

Error analysis of the results of the dictionary-based statistical approach.

Furthermore, thanks to the dictionary-based approach, ideal dictionary enrichment, without any retraining of the models, has shown to contribute to improve the performance to an F-score of 78.72.

## Conclusion and future work

This paper has demonstrated how to utilize known named entities to achieve better performance in statistical named entity recognition. We took a two-step approach where sentences are first tokenized and tagged based on a biomedical dictionary that consists of general English words and about 1.3 million protein names. Then, a statistical sequence labelling step predicted protein names that are not listed in the dictionary and, at the same time, reduced false negatives in the POS/PROTEIN tagging results. The significant benefit of this approach is that a user, not a system developer, can easily enhance the performance by augmenting the dictionary. This paper demonstrated that the state-of-the-art F-score 73.78 on the standard JNLPBA-2004 data set was achieved by our approach. Furthermore, in our dictionary-based statistical NER approach, the upper bound performance using ideal dictionary enrichment, without any retraining of the models, was estimated to an F-score of 78.72.

Our future work includes applying the dictionary-based statistical NER approach to other NE categories, such as metabolite names. Furthermore, it will be of great interest to theoretically and empirically analyze the effect of dictionary enrichment to performance improvement.

## Competing interests

The authors declare that they have no competing interests.

## Authors' contributions

Every author contributed to the design and editing of the manuscript. YS carried out the study and wrote the paper. YT contributed to the methodological design and the feature engineering. The overall supervision was conducted by JM and SA. All authors read and approved the final manuscript.

## References

[B1] Ananiadou S, McNaught J, (eds) (2006). Text Mining for Biology and Biomedicine. Artech House, London.

[B2] Lafferty J, McCallum A, Pereira F (2001). Conditional random fields: probabilistic models for segmenting and labelling sequence data. Proceedings of the Eighteenth International Conference on Machine Learning (ICML-2001).

[B3] Baum LE, Petrie T (1966). Statistical inference for probabilistic functions of finite state Markov chains. The Annals of Mathematical Statistics.

[B4] McCallum A, Freitag D, Pereira F (2000). Maximum entropy Markov models for information extraction and segmentation. Proceedings of the Seventeenth International Conference on Machine Learning.

[B5] Kim J-D, Ohta T, Tsuruoka Y, Tateisi Y (2004). Introduction to the Bio-Entity Recognition Task at JNLPBA. Proceeding of the Joint Workshop on Natural Language Processing in Biomedicine and its Applications (JNLPBA-2004).

[B6] Kim JD, Ohta T, Tateisi Y, Tsujii J (2003). GENIA corpus – semantically annotated corpus for bio-textmining. Bioinformatics.

[B7] Kudo T, Yamamoto K, Matsumoto Y (2004). Applying Conditional Random Fields to Japanese Morphological Analysis. Proceedings of Empirical Methods in Natural Language Processing (EMNLP).

[B8] MeCab. http://sourceforge.net/project/showfiles.php?group_id=177856.

[B9] Aoe J (1989). An Efficient Digital Search Algorithm by Using a Double-Array Structure. IEEE Transactions on Software Engineering.

[B10] MedPost. ftp://ftp.ncbi.nlm.nih.gov/pub/lsmith/MedPost.

[B11] Part-of-Speech Tagging Guidelines for the Penn Treebank Project. ftp://ftp.cis.upenn.edu/pub/treebank/doc/tagguide.ps.gz.

[B12] BioThesaurus. http://pir.georgetown.edu/iprolink/biothesaurus.

[B13] Plos Biology Journals. http://biology.plosjournals.org.

[B14] TreeTagger. http://www.ims.uni-stuttgart.de/projekte/corplex/TreeTagger/DecisionTreeTagger.html.

[B15] Tjong Kim Sang EF, Veenstra J (1999). Representing Text Chunks. Proceedings of the Ninth Conference of the European Chapter of the Association for Computational Linguistics (E-99); Bergen, June 8 – 12, 1999.

[B16] Franzen K, Eriksson G, Olsson F, Asker L, Liden P, Koster J (2002). Protein names and how to find them. International Journal of Medical Informatics.

[B17] Fukuda K, Tsunoda T, Tamura A, Takagi T (1998). Toward information extraction: identifying protein names from biological papers. Proceedings of Pacific Symposium on Biocomputing.

[B18] Collier N, Nobata C, Tsujii J (2000). Extracting the Names of Genes and Gene Products with a Hidden Markov Model. Proceedings of the 18th International Conference on Computational Linguistics (COLING'2000); Saarbrucken.

[B19] Kazama J, Makino T, Ohta Y, Tsujii J (2002). Tuning Support Vector Machines for Biomedical Named Entity Recognition. Proceeding of ACL-2002 Workshop on Natural Language Processing in the Biomedical Domain.

[B20] Lee KJ, Hwang YS, Rim HC (2003). Two-Phase Biomedical NE Recognition based on SVMs. Proceedings of ACL 2003 Workshop on Natural Language Processing in Biomedicine; Sapporo.

[B21] Okanohara D, Miyao Y, Tsuruoka Y, Tsujii J (2006). Improving the Scalability of Semi-Markov Conditional Random Fields for Named Entity Recognition. Proceedings of the Forty fourth Annual Meeting of the Association for Computational Linguistics (ACL-2006); Sydney.

[B22] Tanabe L, Wilbur WJ (2002). Tagging Gene and Protein Names in Biomedical Text. Bioinformatics.

[B23] Tsuruoka Y GENIA Tagger 3.0. http://www-tsujii.is.s.u-tokyo.ac.jp/GENIA/tagger.

[B24] Yamamoto K, Kudo T, Konagaya A, Matsumoto Y (2004). Use of morphological analysis in protein name recognitionstar. Journal of Biomedical Informatics.

[B25] Tsai TH, Sung CL, Dai HJ, Hung HC, Sung TY, Hsu WL (2006). NERBio: using selected word conjunctions, term normalization, and global patterns to improve biomedical named entity recognition. BMC Bioinformatics.

[B26] Zhou GD, Su J (2004). Exploring Deep Knowledge Resources in Biomedical Name Recognition. Proceedings of the Joint Workshop on Natural Language Processing of Biomedicine and its Applications (JNLPBA-2004).

[B27] Kim S, Yoon J (2007). Experimental Study on a Two Phase Method for Biomedical Named Entity Recognition. IEICE Transactions on Informaion and Systems.

[B28] Finkel J, Dingare S, Nguyen H, Nissim M, Sinclair G, Manning C (2004). Exploiting context for biomedical entity recognition: from syntax to the Web. Proceedings of the Joint Workshop on Natural Language Processing in Biomedicine and its Applications (JNLPBA-2004).

[B29] Settles B (2004). Biomedical Named Entity Recognition Using Conditional Random Fields and Novel Feature Sets. Proceeding of the Joint Workshop on Natural Language Processing in Biomedicine and its Applications (JNLPBA-2004).

[B30] Rössler M (2004). Adapting an NER-System for German to the Biomedical Domain. Proceeding of the Joint Workshop on Natural Language Processing in Biomedicine and its Applications (JNLPBA-2004).

[B31] Park K-M, Kim S-H, Lee D-G, Rim H-C (2004). Boosting Lexical Knowledge for Biomedical Named Entity Recognition. Proceeding of the Joint Workshop on Natural Language Processing in Biomedicine and its Applications (JNLPBA-2004).

[B32] Song Y, Kim E, Lee GG, Yi B (2004). POSBIOTM-NER in the shared task of BioNLP/NLPBA. Proceedings of the Joint Workshop on Natural Language Processing in Biomedicine and its Applications (JNLPBA-2004).

